# Cystic Dysplasia of the Rete Testis: Case Report and Systematic Review of the Literature

**DOI:** 10.3389/fped.2022.898038

**Published:** 2022-05-18

**Authors:** Giorgia Contini, Simone Frediani, Valerio Pardi, Francesca Diomedi-Camassei, Alessandro Inserra

**Affiliations:** ^1^General and Thoracic Pediatric Surgery Unit, Bambino Gesù Children's Hospital, Istituto di Ricovero e Cura a Carattere Scientifico (IRCCS), Rome, Italy; ^2^Pathology Unit, Department of Laboratories, Bambino Gesù Children's Hospital, Istituto di Ricovero e Cura a Carattere Scientifico (IRCCS), Rome, Italy

**Keywords:** CDRT, cystic dysplasia, rete testis, testicle, children, testicular mass

## Abstract

Cystic dysplasia of the rete testis (CDRT) is a rare cause of testicular masses in children. The pathogenesis of this malformation remains unclear. It is often associated with other genitourinary anomalies, commonly presenting as agenesis or dysplasia of the ipsilateral kidney. A case involving a 9-year-old boy with a testicular lesion and ipsilateral renal agenesis, who was diagnosed with CDRT after histological examination, is reported. In addition, a systematic review of the literature was performed to better understand this pathology to design the most appropriate treatment and follow-up strategy for patients with CDRT.

## Introduction

Cystic dysplasia of the rete testis (CDRT) is a rare cause of testicular masses in children. It was first described by Leissring and Oppenheimer in 1973 as a rare benign testicular lesion (1). CDRT is characterized by irregular cystic spaces lined by cuboidal epithelium in the mediastinum or rete testes (2). It is often associated with genitourinary tract anomalies, primarily with renal agenesis (3). This malformation is likely the result of a disorder(s) in the connection between the mesonephric duct and germinal epithelium and represents a diagnostically challenging condition in the pediatric population (1). The purpose of this study was to investigate CDRT in a 9-year-old boy with a right testicular lesion and ipsilateral renal agenesis. Furthermore, we performed a systematic review of the literature to better understand this pathology to design the most appropriate treatment and follow-up strategy for patients with CDRT.

## Case Report

**Figure 1:**
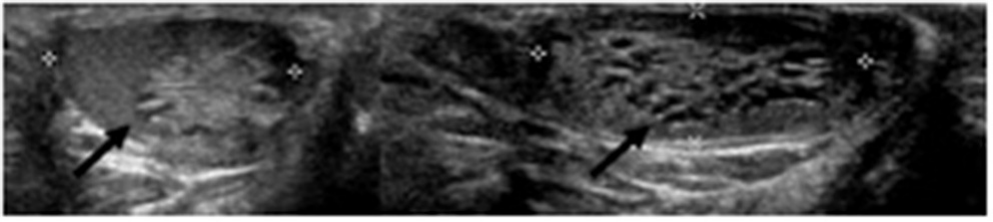
Pre-operative ultrasound of present case. Cysts in the right mediastinum testis (black arrow).

**Figure 2:**
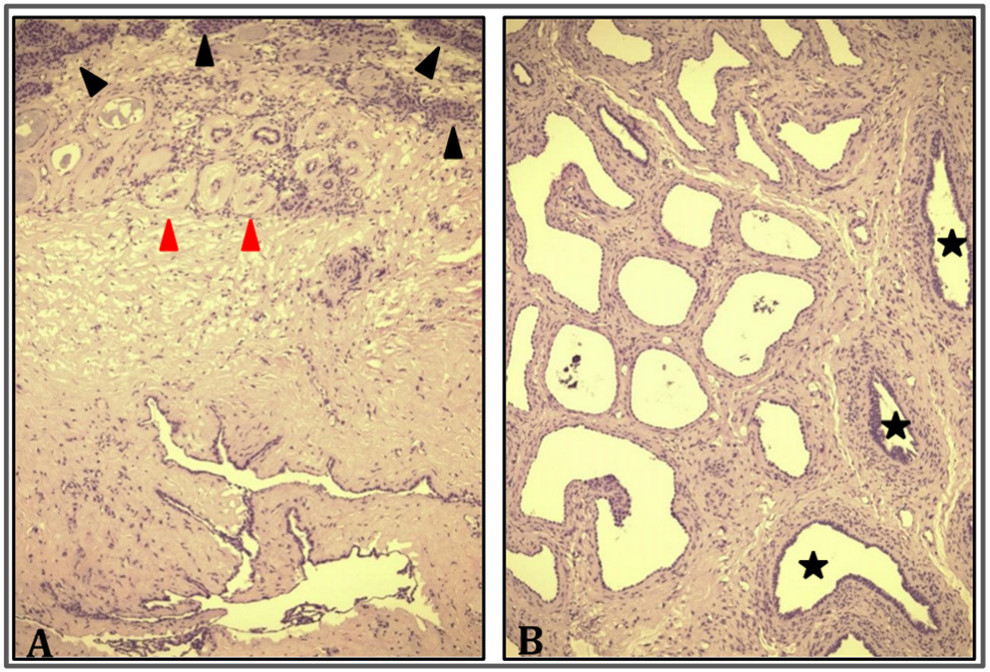
Cystic dysplasia of rete testis. **(A)** Rete testis consisting of irregularly ectasic spaces lined by cuboidal epithelium and surrounded by fibrous stroma (bottom); testicular parenchyma (top) is characterized by prebuberal (black arrowhead) and atrophic seminiferous tubules (red arrowhead) (HE 10×). **(B)** Dilatation and irregularity of rete testis was global and reached the epididymis (black stars) (HE 20×).

A 9-year-old boy with a right retractile testicle underwent testicular ultrasonography, which revealed a testicular lesion and, accordingly, was referred to the authors' hospital. On physical examination, the testicles were in the scrotum, with normal volume and consistency. The right testicle exhibited a palpable upper pole lesion. Testicular ultrasound examination was repeated, revealing a circumscribed area, measuring 20 × 10 × 9 mm, containing several minuscule cysts of varying sizes in the right mediastinal testis (Figure 1). The lesion did not appear vascularised on color Doppler ultrasound and was surrounded by normal testicular parenchyma; in addition, the left testicle was normal. Screening for associated urinary anomalies was performed using abdominal ultrasound and magnetic resonance imaging (MRI). The right kidney was not visualized on abdominal ultrasound, which suggested right renal agenesis, whereas the left kidney and bladder appeared to be normal. Moreover, MRI confirmed right renal agenesis and revealed enlargement of the right epididymis with an extended area of altered signal (16.5 × 9.5 × 12 mm), hypointensity on T1, and hyperintensity on diffusion-weight imaging sequences, with no contrast uptake and without calcific or fat images. Markers for testicular tumors, including alpha-fetoprotein, human chorionic gonadotropin, and lactate dehydrogenase, were within normal limits. Considering the ultrasound characteristics of the lesion associated with renal agenesis, CDRT was suspected, and a conservative treatment strategy was chosen. The patient was followed-up with periodic clinical and ultrasonographic examinations. Testicular ultrasound performed on the lesion 3 and 5 months later revealed no variations in morphology, size, or structural characteristics. Surgical exploration was performed using the inguinal approach to exclude possible malignancy. Intraoperatively, the testicular parenchyma was mostly substituted with spongiform tissue; accordingly, biopsy of the right testicle and epididymis was performed. Histological examination revealed irregular cystic spaces located in the mediastinum of the testis, displacing the testicular parenchyma. The cysts were lined with flattened cuboidal epithelium and separated by fibrous septa. No germ cell tumors were identified (Figure 2). Histological examination, along with renal agenesis, confirmed the diagnosis of CDRT. Four months after surgery, testicular ultrasound revealed no change in the right testicular lesion. One year later, testicular ultrasound was repeated, and the lesion exhibited the same morphological characteristics, with no internal blood flow, although its dimensions increased to 33 × 12 × 27 mm.

At the last follow-up-−28 months after surgery—the patient was in good general condition. Testicular ultrasound revealed a lesion with similar dimensions (35 mm × 16 mm × 16 mm), with unchanged parenchymal characteristics. Tumor marker levels were also determined and remained normal. Finally, it was decided to continue ultrasound follow-up every year due to the benign nature of the lesion.

## Discussion

CDRT is a rare, benign cause of testicular masses in the pediatric population. The differential diagnosis of CDRT includes other intrascrotal pathologies, including hydrocele, hernia, other benign and malignant testicular masses, and testicular torsion. All cystic or multicystic testicular lesions must be considered, including simple intratesticular cysts, epidermoid cysts, tunica albuginea cysts, testicular teratomas or lymphomas, juvenile granulosa cell tumors, gonadal stromal tumors, and cystic lymphangiomas. Ultrasonography can be used for the initial diagnosis and differentiation of these diseases (4).

CDRT has a histological and sonographic appearance similar to tubular ectasia of the rete testis, a benign polycystic testicular disorder caused by obstruction in the epididymis or efferent ductulus in the adult population. This condition is often bilateral and is usually not associated with urinary abnormalities (5). In addition, CDRT is usually unilateral, with no predominance on either side and a mean age at presentation of approximately of 6 years, as reported by Jeyaratnam et al. (6).

CDRT usually manifests as painless scrotal swelling. It is frequently associated with genitourinary anomalies, ipsilateral renal agenesia, and multicystic dysplasia of the kidney (3). Nevertheless, the exact pathogenesis of CDRT remains unclear. Leissring and Oppenheimer suggested that the lack of connection between the mesonephric duct and germinal epithelium at the level of the rete testis leads to progressive degeneration of the mediastinum testis into small cysts. This hypothesis could also explain urinary tract abnormalities that are frequently associated with CDRT. The mesonephric duct originates from the ureteral bud, which eventually forms the kidney (1). In addition, Nistal et al. proposed another hypothesis involving the over-secretion of fluid in immature seminiferous tubules with no lumen. Spontaneous regression of the cysts could be explained by progressive canalization of the tubules during childhood (2).

Owing to the rarity and importance of CDRT, the aim of the present study was to describe a case of CDRT in a 9-year-old child and to perform a systematic review of the literature on which to base the treatment and follow-up of this pathology. Accordingly, a systematic literature review was performed in accordance with the Preferred Reporting Items for Systematic Reviews and Meta-Analyses (i.e., “PRISMA”) guidelines. Eligible studies included those that investigated CDRT and were published as full-text articles by indexed journals in the Cochrane, MEDLINE via PubMed, Embase, or Scopus databases. Keywords used in the literature search included “cystic dysplasia rete testis” and their MeSH terms in any possible combination. Only articles published in English with available abstracts were included, with no limits on publication date. The reference lists of relevant studies were screened to identify other potentially eligible studies. The search was repeated up to November 30, 2021.

Editorial comments, letters to the editor, studies involving animals, adults, unpublished reports, studies on deceased fetuses, abstracts from scientific meetings, and book chapters were excluded from the review.

**Figure 3:**
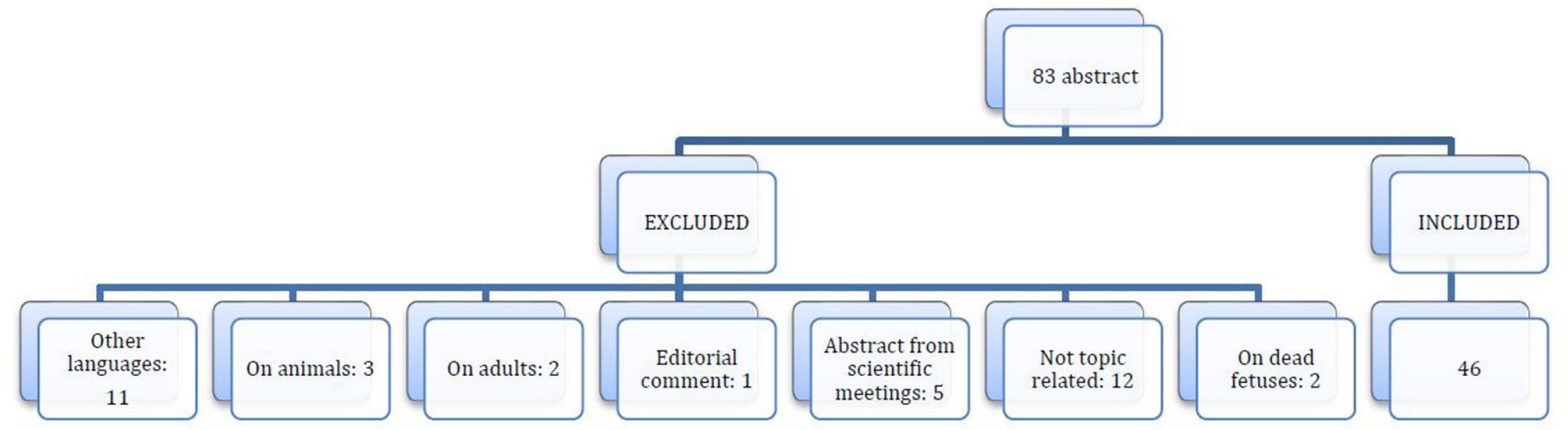
PRISMA flow diagram.

The risk of bias for observational studies was appraised using the methodological index for non-randomized studies (MINORS) (7). Risk of bias was assessed by two reviewers (GC and SF), who extracted data from the included studies. Any discordance was resolved by consensus with the third author (VP). For each study included in the analysis, the following data were extracted: demographic features; number of patients; clinical features; treatment performed; and follow-up. After eliminating duplicates, the initial literature search retrieved 83 potential studies, as shown in the PRISMA flow diagram (Figure 3). After applying the inclusion and exclusion criteria, 46 articles, including 65 patients (66 including our case report), were selected (Table 1).

**Table 1:** Characteristics of studies included in the review.

**References**	**Type of study**	**Nationality**	**Age at diagnosis (months)**	**Side**	**Associated malformations**	**Clinical presentation**	**Diagnosis**	**Tumor markers (bHCG; aFP; LDH)**	**Treatment**	**Duration follow-up**	**Evolution last follow-UP**
Present case	Case report	Italy	108	R	IRA	Right asymptomatic testicular mass in retractile testicle	US; RMI	Normal	Biopsy	16 m	Persistence
Pizzuti et al. (4)	Case report	Italy	18	L	NO	Left painless scrotal swelling	US	Normal	Observation (clinical and US examinations)	6 m	Regression
Helman et al. (8)	Case report	United States	36	R	IRA; IESV	Right painless scrotal swelling	US; RMI	Normal	Observation (clinical and US examinations)	NR	NR
Fuchs et al. (9)	Case report	United States	48	NR	NR	NR	NR	NR	Testicular- sparing surgery	48 m	Disease-free
Friend et al. (10)	Case report	Australia	11	L	NO	Non-palpable left testicle since birth	Laparoscopy	NR	Orchiectomy	NR	NR
Gelas et al. (11)	Case series	France	Neonate	R	NO	Antenatal US (enlarged testicle)	US	Normal	Observation (clinical and US examinations)	116 m	Regression
			Neonate	L	NO	Left painless scrotal swelling	US	Normal	Observation (clinical and US examinations)	16 m	Regression
			Neonate	L	NO	Left painless scrotal swelling	US	Normal	Observation (clinical and US examinations)	24 m	Regression
Delto et al. (12)	Case report	United States	216	L	IRA; IESV	Left testicular pain	US; RMI; CT	Normal	Observation (clinical and US examinations)	NR	NR
Liniger et al. (13)	Case report	Switzerland	144	R	IRA	Right painless scrotal swelling	US	Normal	Testicular sparing surgery	45 m	Disease-free
Emam et al. (14)	Case report	Saudi Arabia	2	R	NO	Right cryptorchidism	US; RMI	Normal	Orchiectomy	NR	NR
Poupalou et al. (15)	Case report	France	17	R	Ipsilat. ectopic MCDK	Right painless scrotal swelling	US	Normal	Biopsy	NR	NR
Butler et al. (16)	Case report	United States	48	L	IRA	Left painless scrotal swelling	NR	NR	Biopsy	108 m	Regression
Jeyaratnam et al. (6)	Case report	United Kingdom	18	R	IRA	right painless scrotal swelling	US	Normal	Observation (clinical and US examinations)	138 m	Regression
Meiràs et al. (17)	Case report	Spain	1	R	NO	Right painless scrotal swelling	US	Normal	Orchiectomy	12 m	NR
McGee et al. (18)	Case report	United States	180	R	Ipsilat. atrophic kidney; ipsilat. ectopic hydroureter; IESV	Right testicular pain	US; CT	NR	Observation (clinical and US examinations)	NR	Persitence
Mc New et al. (19)	Case report	United States	2	R	NO	Right asymptomatic testicular mass	US	Normal	Orchiectomy	NR	NR
Park et al. (20)	Case report	United Sates	156	R	VATER association; IRA	right painless scrotal swelling	US	NR	NR	NR	NR
Smith et al. (21)	Case report	United States	96	L	Ipsilat. MCDK	Left testicular pain	US	Normal	Testicular sparing surgery	0.25 m	Disease-free
Taskinen et al. (22)	Case report	Finland	37	NR	Ipsilat. MCDK	Painless scrotal swelling	US	Normal	Testicular- sparing surgery	42 m	Disease-free
Bath et al. (23)	Case report	India	24	R	NO	Right painless scrotal swelling	US	NR	Orchiectomy	NR	NR
Nanni et al. (24)	Case report	Italy	120	R	IRA	Right scrotal swelling after orchidopexy	US	Normal	Orchiectomy	NR	NR
Kajo et al. (25)	Case report	Slovakia	48	L	IRA	Left asymptomatic testicular mass	US	NR	Orchiectomy	20 m	NR
Pohl et al. (26)	Case series	United States	Under 12 years	NR	NR	NR	NR	NR	NR	NR	NR
			Under 12 years	NR	NR	NR	NR	NR	NR	NR	NR
Cottone et al. (27)	Case report	United States	60	R	IRA	Right painless scrotal swelling	US	Normal	Orchiectomy	24 m	NR
Thomas et al. (28)	Case series	United States	108	L	Ipsilat. MCDK; VATER association	Left painless scrotal swelling	US	Normal	Surgical exploration without resection	48 m	Regression
			NR	NR	IRA	NR	NR	Normal	NR	NR	NR
Burns et al. (29)	Case report	United States	144	R	Controlat. renal agenesis; ipsilat. hydronephrosis	Urinary tract infection; right painless scrotal swelling	US	Normal	Testicular- sparing surgery	12 m	Recidive
Eberli et al. (3)	Case report	Switzerland	108	R	IRA	right painless scrotal swelling	US; RMI	Normal	Testicular- sparing surgery	24 m	Recidive
Camassei et al. (30)	Case series	Italy	Neonate	L	Bilat. cryptorchidism; left inguinal hernia; ipsilat. MCDK	Evidence of enlarged left testis during orchidopexy	US	NR	Biopsy; orchiectomy (after 6 mm)	18 m	NR
			Neonate	L	NO	Left testis torsion	US	NR	Orchiectomy	NR	NR
Emir et al. (31)	Case report	Turkey	6	R	Hypospadia; unilateral cryptorchidism; bilat. inguinal hernia; hypoplastic bladder; urethral obstruction	Bilateral hydroureteronephrosis	US	Normal	Orchiectomy	NR	NR
Piotto et al. (32)	Case series	Australia	108	L	IRA	Left painless scrotal swelling	US	NR	Orchiectomy	NR	NR
			96	L	IRA	Retractile right testis; left painless scrotal swelling	US	NR	Testicular- sparing surgery; redo for recidive (after 1 y)	24 m	Recidive
Koumanidou et al. (33)	Case series	Greece	72	R	Ipsilat. MCDK	Right painless scrotal swelling	US	NR	Orchiectomy	NR	NR
			12	L	NO	Left painless scrotal swelling	US	NR	Orchiectomy	NR	NR
Garrett et al. (34)	Case report	United States	Neonate	L	IRA	Left painless scrotal swelling	US	NR	orchiectomy	NR	NR
Toffolutti et al. (35)	Case series	Italy	96	L	NO	Left testicular pain	US	NR	Observation (clinical and US examinations)	24 m	Persistence
			60	Bilateral	Right testicular atrophy	Right pain testicular swelling	US	NR	Observation (clinical and US examinations)	18 m	Persistence
			144	Bilateral	Right ureteral duplication	Right painless scrotal swelling	US	NR	Observation (clinical and US examinations)	16 m	Persistence
Noh et al. (36)	Case sieries	United States	66	L	IRA	Left pain testicular swelling	US	NR	Testicular- sparing surgery; redo for recidive (after 4 m)	14 m	Disease-free
			108	L	Ipsilat. MCDK; VATER association	Left painless scrotal swelling	US	NR	Surgical esploration without resection	25 m	Persistence
Levin et al. (37)	Case report	United States	84	L	Ipsilat. MCDK	Left painless scrotal swelling	US; CT	Normal	Orchiectomy	NR	NR
Ngai et al. (38)	Case report	Hong Kong	48	R	IRA; anorectal anomaly	Right painless scrotal swelling	US	Normal	Orchiectomy	NR	NR
Robson et al. (39)	Case report	United States	36	L	Ipsilat. MCDK	Left painless scrotal swelling	US	NR	Orchiectomy	NR	NR
Bonnet et al. (40)	Case report	France	120	R	IRA	Right painless scrotal swelling	US	NR	Orchiectomy	NR	NR
Wojcik et al. (41)	Case series	United States	48	R	IRA	Right painless scrotal swelling	US	Normal	Orchiectomy	48 m	NR
			84	L	Ipsilat. renal dysplasia	Left hydrocele	US	Normal	Testicular- sparing surgery	30 m	Disease-free
			72	R	IRA	Right painless scrotal swelling	US	Normal	Orchiectomy	45 m	NR
			Neonate	R	Ipsilat. hypoplastic kidney; left hydronephrosis; urethral atresia; bilat. crhyptorchidism; megaphallus	Urinary retention; right abdominal mass;	US	Normal	Orchiectomy	78 m	NR
			96	R	IRA	Right painless scrotal swelling	US	Normal	Testicular sparing surgery; orchiectomy for recidive (after 8 m)	46 m	NR
			144	R	IRA	Right painless scrotal swelling	US	NR	Orchiectomy	78 m	NR
Zaragoza et al. (42)	Case report	United States	48	R	IRA	Right painless scrotal swelling	US	Normal	Orchiectomy	NR	NR
Simoneaux (43)	Case report	United States	Neonate	L	Ipsilat. MCDK	Left painless scrotal swelling	US	NR	Orchiectomy	NR	NR
Loo et al. (44)	Case series	Australia	72	L	Ipsilat. renal atrophy	Left painless scrotal swelling	US	NR	Orchiectomy	NR	NR
			24	L	Left inguinal hernia	Left painless scrotal swelling	US	NR	Orchiectomy	NR	NR
			60	R	IRA	Penis pain; right painless scrotal swelling	US	NR	Orchiectomy	NR	NR
Glantz et al. (45)	Case report	United States	54	R	IRA	Right hydrocele	US	NR	Orchiectomy	NR	NR
Keetch et al. (46)	Case report	United States	144	R	IRA	Right painless scrotal swelling	US	NR	Orchiectomy	NR	NR
Tesluk et al. (47)	Case report	United States	Neonate	L	Right MCDK; urethral stricture; bilat. hydroureters; hypoplastic lungs; testis in the abdomen	Bilat. cryptorchidism (died at 8 d)	Autopsy	NR	NA	NA	NA
Nistal et al. (2)	Case series	Spain	Neonate	Bilateral	Potter's syndrome; bilat. renal dysplasia; testis in the abdomen	Bilat. Cryptorchidism (died at 8 d)	autopsy	NR	NA	NA	NA
			Neonate	Bilateral	Intracranial hemorrhage; bilat. Pulmonary atelectasis; interauricular communication	NR (died at 8 d)	autopsy	NR	NA	NA	NA
			108	L	Bilat. cryptorchidism, adrenal choristoma	Bilat. cryptorchidism	Intraoperative	NR	Orchiectomy	NR	NR
Fisher et al. (48)	Case report		120	L	IRA	Left painless scrotal swelling	Clinical	NR	Testicular sparing surgery	15 m	Disease-free
Leissring et al. (1)	Case report	United States	48	R	IRA	Chronic painless right scrotal swelling	Clinical	NR	Orchiectomy	NR	NR

*NR, not reported; NA, not applicable; IRA, ipsilateral renal agenesis; MCDK, multicystic dysplasia of the kidney; IESV, ipsilateral enlarged seminal vesicle*.

Results of this study confirmed the many characteristics of this disease and highlighted further interesting aspects.

Data are reported as mean or rate, and were analyzed using GraphPad Prism version 4.00 (GraphPad Software, San Diego, CA; http://www.graphpad.com) for Windows (Microsoft Corporation, Redmond, WA, USA).

The age at presentation ranged from birth to 18 years, with a mean of 5.2 years (62.5 months). Only two cases involving adults, 23 and 63 years of age, respectively, have been reported and were excluded from this study.

CDRT was usually unilateral [*n* = 62 (93.9%)], while bilateral lesions were reported in only four (6%) patients. In addition, CDRT exhibited no predominance on either side [right, *n* = 30 (45.5%); and left, *n* = 27 (41%)]. The most frequent clinical presentation was painless scrotal swelling [*n* = 40 (60.6%)], followed by penile pain (*n* = 1), asymptomatic testicular mass [*n* = 3 (4.5%)], testicular pain [*n* = 6 (9%)], and testicular torsion [*n* = 1 (1.5%)]. In one (15%) case, the lesion was suspected after antenatal ultrasound, while in four (6%), the lesion was discovered after diagnostic examinations for an undescended testicle. Two patients with non-palpable testes at birth died of comorbidities, and the diagnosis was made after autopsy. Another patient with testes in the scrotum was born in a tenuous clinical condition and received an autopsy diagnosis (2). Two (5.7%) patients presented with hydrocele, one (1.5%) with bilateral hydroureteronephrosis, and one (1.5%) with urinary retention, with a right abdominal mass for urethral atresia. Clinical presentation was not described in four (6%) patients.

CDRT was frequently associated with urogenital system anomalies [*n* = 50 (75.8%)], with the most common being ipsilateral renal agenesis [*n* = 28 (50%)], whereas contralateral renal agenesis was found in only one (1.5%) case. Other anomalies were also reported, including multicystic dysplasia of the kidney [*n* = 11 (16.6%)], one of which was contralateral, hypoplastic/atrophic kidney [*n* = 3 (4.5%)], enlarged seminal vesicle [*n* = 3 (4.5%)], renal dysplasia [*n* = 2 (3%)], duplication of ureters [*n* = 2 (3%)], pyeloureteral stenosis [*n* = 2 (3%)], hydroureter [*n* = 2 (3%)], urethral stricture [*n* = 2 (3%)], hypospadia [*n* = 1 (1.5%)], and megaphallus [*n* = 1 (1.5%)]. Other malformations associated with CDRT included Vater association [*n* = 3 (4.5%)], Potter's syndrome [*n* = 1 (1.5%)], and anorectal malformation [*n* =1 (1.5%)].

Ultrasound is the gold standard for the diagnosis and follow-up of CDRT. MRI can be used as a complementary examination (14). In our review, testicular ultrasound was used in 54 (81.8%) patients. The description of ultrasound imaging for CDRT was similar in all reported cases. The affected testicle was usually enlarged, with a mass of small cysts (2–8 mm in size) in the rete testis. The surrounding testicular tissue and epididymis were normal but compressed. As an exception, Robson et al. reported a case of CDRT in which ultrasonography revealed a solid lesion (39). When cysts are extremely small, they can appear as echogenic foci, mimicking testicular microlithiasis (3).

Markers of testicular tumors (i.e., alpha-fetoprotein, beta-human chorionic gonadotropin, and lactate dehydrogenase) were normal when tested [*n* = 30 (45.5%)] in all reported cases of CDRT. In 45 (68%) patients, histological examinations were available and were similar in all cases reported.

Histologically, CDRT is typically characterized by a multicystic lesion primarily located in the mediastinum testis. The cystic spaces were separated by connective tissue lined by cuboidal cells. Cysts usually differ in shape and size (ranging from several millimeters) (3). Moreover, cystic spaces express keratin and vimentin in a cytoplasmic pattern, as well as epithelial membrane antigens, such as that of the ductular epithelium of the mediastinum testis (45).

There are no clear diagnostic criteria for CDRT. Levin et al. suggested that CDRT can be suspected if the lesion is well-circumscribed with normal surrounding parenchyma and is composed of multiple small cysts revealed on ultrasound, if tumor markers are normal, and if there are associated mesonephric anomalies (37). If these criteria are fulfilled, as in the present case, open biopsy of the lesion is not immediately necessary to confirm the diagnosis. No standard treatment has been defined.

Previously, orchiectomy was the treatment of choice (1). Recently, because of better understanding of the benign nature of this pathology, a conservative approach was proposed, such as testicular-sparing surgery or observation (11). Poupalou et al. proposed testicular-sparing surgery for large lesions at diagnosis or enlarging lesions under observation (15). More specifically, we found that in all reported cases of CDRT, 32 (48.5%) were treated with orchiectomy as a definitive treatment. In 11 (16.7%) cases, testicular-sparing surgery with excision of the lesion was performed with preservation of the normal testicular parenchyma. Biopsy was performed in three (4.5%) patients. Only two (3%) patients underwent surgical exploration without biopsy or excision of the lesion. In 11 (16.7%) patients, an observational approach (without biopsy) was adopted, and the patients were monitored with periodic clinical and ultrasonographic follow-up. Follow-up data after orchiectomy were available for only nine cases. No recurrence was reported after a mean follow-up of 41 months. In contrast, of the 11 patients treated using the testicular-sparing approach, five (45.5%) experienced recurrence of the cyst after a median of 12 months, confirming the importance of radical treatment in conservative surgery, maintaining a safety margin between the removed mass and the healthy parenchyma.

Different treatments for recurrence have been used. The testicular-sparing approach was chosen in two patients, one of which experienced another recurrence after the second surgery. Orchiectomy was performed in two patients without evidence of recurrence at follow-up, while in one case, reoperation was not performed. Of the 16 patients who did not undergo orchiectomy or testicular-sparing surgery, regression of the lesion was reported in seven (43.8%). These patients were followed up with cyclical ultrasound until complete resolution of CDRT after a median of 59.6 months from diagnosis. Remarkably, four (57.1%) of these patients did not have an associated malformation. Helman et al. recently suggested a diagnostic and management algorithm reserving surgical intervention only for cases in which the diagnosis was unclear. Yearly scrotal ultrasound was proposed for patients who met the criteria for CDRT (8). When non-surgical management is chosen, close follow-up during the first few months after diagnosis is mandatory (11). However, surgical biopsy and histological confirmation are indispensable for definitive diagnosis and to rule out malignant cystic testicular lesions, especially when there is no ultrasound regression of the lesion.

In conclusion, CDRT is a rare diagnosis of testicular masses in the pediatric population. It is usually unilateral with no predominance on either side, and the mean age at presentation is ~5–6 years. CDRT usually manifests as painless scrotal swelling. It is frequently associated with genitourinary anomalies, particularly ipsilateral renal agenesis; however, its pathogenesis remains unclear. Ultrasound is the gold standard for the diagnosis and follow-up of CDRT, although there are no clear diagnostic criteria for CDRT. However, it can be suspected if the lesion is well-circumscribed with normal surrounding parenchyma and is composed of multiple small cysts on ultrasound, if tumor markers are normal, or if there are associated mesonephric anomalies. When non-surgical management is chosen, close follow-up during the first few months after diagnosis is mandatory. Surgical biopsy and histological confirmation are indispensable for definitive diagnosis and for ruling out malignant cystic testicular lesions, especially when there is no ultrasound evidence of regression of the lesion.

The principal limitation of this systematic review was the presence of only case report and case series with short to intermediate follow up in the international literature. Moreover, not all data were reported in the analyzed studies.

## Data Availability Statement

The datasets for this article are not publicly available due to concerns regarding participant/patient anonymity. Requests to access the datasets should be directed to the corresponding author.

## Ethics Statement

All procedures performed in studies involving human participants were in accordance with the ethical standards of the Institutional and/or National Research Committee and with the 1964 Helsinki Declaration and its later amendments or comparable ethical standards.

## Author Contributions

GC, SF, VP, FD-C, and AI have made substantial contributions to the development of the manuscript, read and approved the version submitted, and share responsibility for the content.

## Conflict of Interest

The authors declare that the research was conducted in the absence of any commercial or financial relationships that could be construed as a potential conflict of interest.

## Publisher's Note

All claims expressed in this article are solely those of the authors and do not necessarily represent those of their affiliated organizations, or those of the publisher, the editors and the reviewers. Any product that may be evaluated in this article, or claim that may be made by its manufacturer, is not guaranteed or endorsed by the publisher.
